# Hevin/Sparcl1 drives pathological pain through spinal cord astrocyte and NMDA receptor signaling

**DOI:** 10.1172/jci.insight.161028

**Published:** 2022-12-08

**Authors:** Gang Chen, Jing Xu, Hao Luo, Xin Luo, Sandeep K. Singh, Juan J. Ramirez, Michael L. James, Joseph P. Mathew, Miles Berger, Cagla Eroglu, Ru-Rong Ji

**Affiliations:** 1 Center for Translational Pain Medicine, Department of Anesthesiology, and; 2 Department of Cell Biology, Duke University Medical Center, Durham, North Carolina, USA.; 3 Department of Biochemistry and Molecular Biology, Virginia Commonwealth University School of Medicine, Richmond, Virginia, USA.; 4 Department of Neurobiology,; 5 Department of Anesthesiology, and; 6 Howard Hughes Medical Institute, Duke University Medical Center, Durham, North Carolina, USA.; 7 Duke Institute for Brain Sciences (DIBS), Durham, North Carolina, USA.

**Keywords:** Neuroscience, Neurological disorders

## Abstract

High endothelial venule protein/SPARC-like 1 (hevin/Sparcl1) is an astrocyte-secreted protein that regulates synapse formation in the brain. Here we show that astrocytic hevin signaling plays a critical role in maintaining chronic pain. Compared with WT mice, hevin-null mice exhibited normal mechanical and heat sensitivity but reduced inflammatory pain. Interestingly, hevin-null mice have faster recovery than WT mice from neuropathic pain after nerve injury. Intrathecal injection of WT hevin was sufficient to induce persistent mechanical allodynia in naive mice. In hevin-null mice with nerve injury, adeno-associated-virus–mediated (AAV-mediated) re-expression of hevin in glial fibrillary acidic protein–expressing (GFAP-expressing) spinal cord astrocytes could reinstate neuropathic pain. Mechanistically, hevin is crucial for spinal cord NMDA receptor (NMDAR) signaling. Hevin-potentiated N-Methyl-D-aspartic acid (NMDA) currents are mediated by GluN2B-containing NMDARs. Furthermore, intrathecal injection of a neutralizing Ab against hevin alleviated acute and persistent inflammatory pain, postoperative pain, and neuropathic pain. Secreted hevin that was detected in mouse cerebrospinal fluid (CSF) and nerve injury significantly increased CSF hevin abundance. Finally, neurosurgery caused rapid and substantial increases in SPARCL1/HEVIN levels in human CSF. Collectively, our findings support a critical role of hevin and astrocytes in the maintenance of chronic pain. Neutralizing of secreted hevin with monoclonal Ab may provide a new therapeutic strategy for treating acute and chronic pain and NMDAR-medicated neurodegeneration.

## Graphical abstract

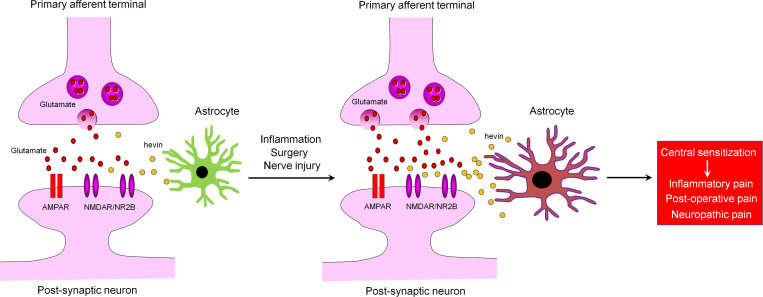

## Introduction

Chronic pain, such as neuropathic pain, is a major health problem worldwide and difficult to treat (1, 2). It is generally believed that maladaptive synaptic plasticity in the spinal cord and brain drives chronic pain (1, 3). Activation of N-Methyl-D-aspartic acid receptors (NMDARs), especially the NR2B subunit (GluN2B), plays a crucial role in injury-induced synaptic plasticity and pain pathogenesis (4, 5). Astrocytes are a major glial cell type in the CNS and critical for maintaining CNS homeostasis, including physiological pain (6–8). Astrocytes make close contacts with synapses, and, therefore, are positioned to regulate synapse formation and synaptic plasticity (9–12). During development, astrocytes promote synapse formation and regulate synaptic connectivity in the CNS through secreted signals, such as thrombospondin-4 (TSP4) (9, 10, 13) or adhesion molecules, such as neuroligins and hepatocyte cell adhesion molecule (11, 14). Multiple types of neurological and neuropsychiatric disorders, such as Alzheimer’s disease and chronic pain, may result from gliopathy of astrocytes (15), leading to an inflammatory and pathological A1-like phenotype (16). Several lines of evidence support an essential role of astrocytes in neuropathic pain development and maintenance (15, 17–21). Following painful nerve injury or chemotherapy-induced neuropathy, astrogliosis is more prominent and persistent than microgliosis in the spinal cord dorsal horn (SDH) (15, 22). Several signaling molecules, such as connexin-43 (Cx43), chemokines (CCL2 and CXCL1), MAP kinases (ERK/JNK), and JAK-STAT3 have been implicated in astrocyte signaling to maintain neuropathic pain (6, 18, 19, 23). Nerve injury upregulates TSP4 in spinal astrocytes and facilitates neuropathic pain via modulation of excitatory synaptic transmission (24). Gabapentin is a widely used drug for treating neuropathic pain through interaction with calcium channel α2δ-1 subunit (Cavα2δ1), but interestingly, Cavα2δ1 has been identified as a thrombospondin receptor and regulates excitatory synaptogenesis (13). TSP4-induced pain can be blocked by gabapentin or Cavα2δ1 knockdown (25). Furthermore, a population of astrocytes in superficial SDH is involved in descending noradrenergic control of mechanical pain (26). However, it is not fully understood how astrocytes regulate synaptic plasticity in pathological pain.

Hevin, short for high endothelial venule protein, also known as SPARC-like 1 (SPARCL1) or synaptic cleft 1 (SC1) (27), is a member of the SPARC family of glycoproteins that regulate cell-matrix interactions. Like TSP4, hevin is an astrocyte-secreted synaptogenic protein (9, 10), but its role in chronic pain is unclear. Using a combination of in vitro and in vivo approaches, we demonstrated that hevin assembles Vesicular Glutamate Transporter 2–positive (VGlut2-positive) thalamocortical synapses by bridging neurexin-1α (Nrx1α), a presynaptic component, and neuroligin-1B (NL1B), a postsynaptic component (9). Nrx1α and NL1B do not interact with each other under normal conditions, but hevin can bridge these 2 adhesion proteins across the synapse through interactions mapped to a specific synaptogenic domain (9). These interactions of hevin are critical for the formation and plasticity of thalamocortical synapses in the developing visual cortex via specific regulation of GluN2B-containing NMDARs (9). In this study, we investigated the role of hevin in physiological, inflammatory, neuropathic, and postoperative pain. We demonstrated that hevin is sufficient and required for inducing central sensitization (synaptic plasticity in the spinal cord pain circuit) and mechanical pain (tactile allodynia) via NMDAR/GluN2B signaling. Our data also show that hevin is secreted in mouse and human CSF under injury conditions, and neutralization of the secreted hevin protein with a monoclonal Ab effectively alleviates neuropathic pain. Furthermore, we demonstrate that re-expression of hevin in spinal cord astrocytes of KO mice lacking hevin is sufficient to reinstate neuropathic pain.

## Results

### Hevin is distinctly required in physiological pain, inflammatory pain, and neuropathic pain.

**Figure 1:**
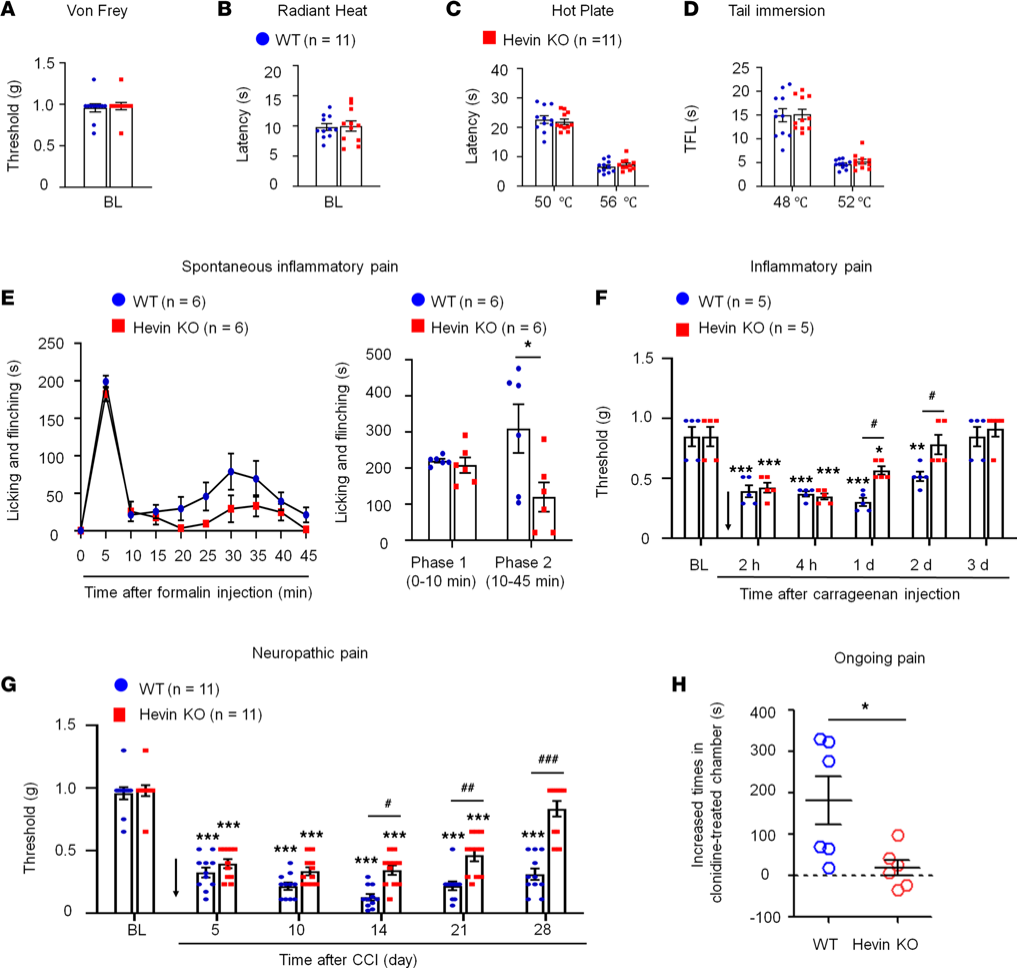
Baseline pain, inflammatory pain, and neuropathic pain in WT and hevin-KO mice. There are no significant differences in mechanical and thermal pain threshold between WT and hevin-KO male mice, as shown in von Frey test (**A**), radiant heat test (**B**), hot plate test (**C**) and tail immersion test (**D**). *P* > 0.05, unpaired Student’s *t* test, *n* = 11 mice/group. (**E**) Formalin-induced acute inflammatory pain was significantly reduced in hevin-KO male mice. Left, time-course of licking and flinching behavior following intraplantar injection of 5% formalin. Right, formalin-induced Phase I (1–10 min) and Phase II (10–45 min) responses. *P < 0.05, 2-way ANOVA followed by Bonferroni’s post hoc test. *n* = 6 mice/group. (**F**) Mechanical allodynia, induced by intraplantar injection of carrageenan, recovered faster in hevin-KO male mice than in WT male mice. Arrow indicates the time of carrageenan injection. *P < 0.05, **P < 0.01, ***P < 0.001, compared with baseline (BL) group; ^#^*P* < 0.05, 2-way ANOVA followed by Bonferroni’s post hoc test. *n* = 5 mice/group. Data shown as mean ± SEM. (**G**) Hevin-KO male mice recovered faster from CCI-induced persistent mechanical allodynia than WT male mice. ***P < 0.001 compared with BL group; ^#^*P* < 0.05, ^##^*P* < 0.01, ^###^*P* < 0.001, 2-way ANOVA followed by Bonferroni’s post hoc test. *n* = 11 mice/group. (**H**) Ongoing pain 3 weeks after CCI in WT and hevin-KO male mice were tested using a 2-chamber CPP. Ongoing pain was present in WT mice but absent in hevin-KO mice following clonidine treatment (10 μg, i.t.). *P < 0.05, unpaired Student’s *t* test, *n* = 6 mice/group. All data are shown as mean ± SEM.

We first set out to assess pain sensitivity in WT and hevin-KO mice in physiological and pathological conditions. Compared with WT control, KO mice exhibited normal baseline mechanical sensitivity as measured in von Frey testing (Figure 1A) and thermal sensitivity in Hargreaves test, hot plate, and tail immersion tests (Figure 1, B–D), suggesting that hevin is dispensable for physiological pain. Next, we examined formalin-induced acute inflammatory pain, which is typically divided into Phase 1 (0–10 min) and Phase 2 (10–45 min). Notably, Phase 2 spontaneous pain is driven by NMDAR-mediated spinal neuron sensitization (central sensitization). We found that formalin-induced Phase 2, but not Phase 1, spontaneous pain was significantly reduced in KO mice (Figure 1E), supporting a role of hevin in central sensitization-mediated pain. Intraplantar carrageenan induced rapid and persistent inflammatory pain that fully recovered in 3 days in WT mice; however, hevin-KO mice showed a faster recovery of this inflammatory pain (Figure 1F). Chronic constriction injury (CCI) of the sciatic nerve induced neuropathic pain, manifested as mechanical allodynia, a reduction in paw withdrawal threshold (PWT), which was maintained at 28 days in WT mice. Strikingly, even though the induction phase of neuropathic pain (5–10 days) was not altered in KO mice compared with WT mice, the CCI-induced mechanical allodynia was fully recovered at 28 days in hevin-KO mice (Figure 1G). We also measured CCI-induced ongoing pain (3 weeks after CCI) using a 2-chamber conditioned place preference (CCP) test (28). The result showed that hevin-KO mice spent less time than WT mice in the analgesic clonidine-treated chamber (Figure 1H), indicating that nerve injury-induced ongoing pain maintenance is reduced in hevin-KO mice. These results show that hevin is required for driving inflammatory and neuropathic pain but dispensable for physiological pain.

### Hevin is sufficient to induce mechanical allodynia in WT mice.

**Figure 2:**
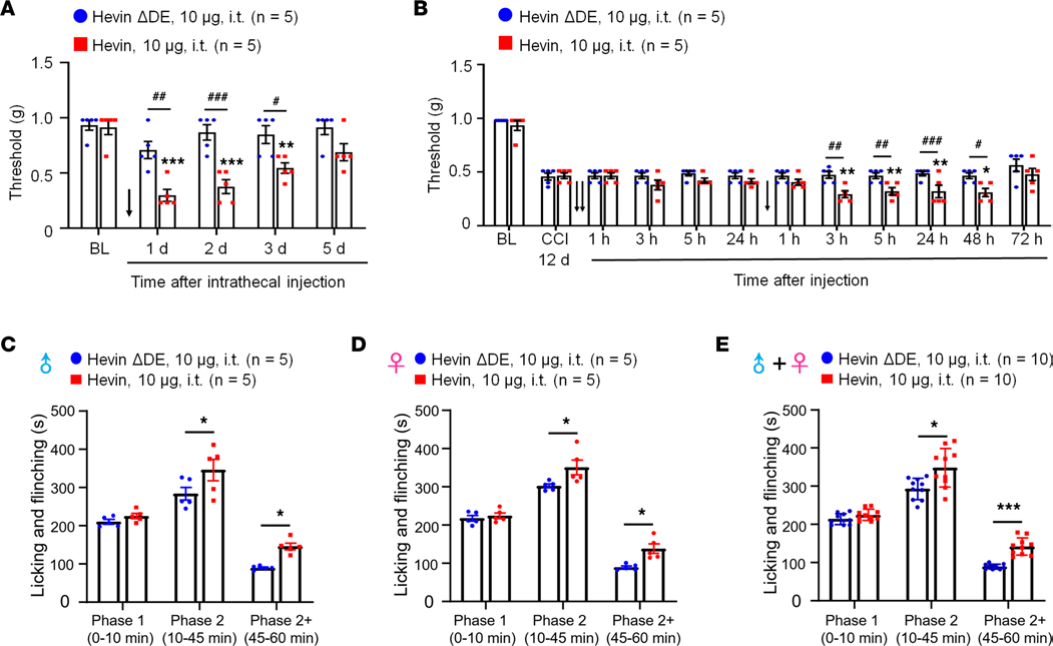
Intrathecal administration of hevin but not hevin-ΔDE (10 μg, i.t.) decreases PWT in naive mice and mice with nerve injury and enhances spontaneous pain by formalin. (**A**) Intrathecal injection of WT hevin but not mutant hevin (hevin-ΔDE) induced mechanical allodynia in naive mice lasting more than 3 days. *n* = 5 mice/group. **P < 0.01, ***P < 0.001 compared with BL group; ^#^*P* < 0.05, ^##^*P* < 0.01, ^###^*P* < 0.001, 2-way ANOVA followed by Bonferroni’s post hoc test. Arrow indicates hevin injection on day 0. (**B**) Repeated intrathecal injections of WT hevin but not mutant hevin (hevin-ΔDE), given at 12 and 13 days after CCI induced further exacerbated mechanical allodynia. *n* = 5 mice/group. *P < 0.05, **P < 0.01, compared with CCI-12d baseline group; ^#^*P* < 0.05, ^##^*P* < 0.01, ^###^*P* < 0.001, 2-way ANOVA followed by Bonferroni’s post hoc test. Arrows indicate the time of hevin injections. (**C**–**E**) Intrathecal injection of WT hevin but not mutant hevin (hevin-ΔDE) enhances formalin-induced licking and flinching on Phase 2 (10–45 min) and Phase 2^+^ (45–60 min) but not Phase 1 (0–10 min) in both male **C** and female **D** mice. *P < 0.05, ***P < 0.001, compared with hevin-ΔDE group, 2-way ANOVA followed by Bonferroni’s post hoc test. All data are shown as mean ± SEM.

Next, we evaluated whether administration of exogenous hevin via intrathecal route to target spinal cord in naive mice would elicit mechanical pain. We also included a hevin mutant which cannot bridge Nrx1a and, thus, NL1B cannot induce synapse formation (hevin-ΔDE, lacking aa 351–440; Supplemental Figure 1; supplemental material available online with this article; https://doi.org/10.1172/jci.insight.161028DS1) as a negative control (9). Intrathecal injection of purified WT hevin (10 μg, intrathecal [i.t.]) but not hevin-ΔDE (10 μg, i.t.) induced robust and persistent mechanical allodynia in naive male mice; this effect lasted for more than 3 days and the mice recovered after 5 days (Figure 2A). We also examined whether hevin could exacerbate neuropathic pain. Intrathecal injections of WT but not hevin-ΔDE, given 12 days after CCI, further enhanced mechanical allodynia in male mice with nerve injury (Figure 2B). Moreover, intrathecal injection of purified WT hevin (10 μg) significantly enhanced formalin-induced spontaneous pain in Phase 2 (10–45 min) in male mice (*P* < 0.05 vs. hevin-ΔDE; Figure 2C), female mice (*P* < 0.05 vs. hevin-ΔDE; Figure 2D), and males and females combined (*P* < 0.05 vs. hevin-ΔDE; Figure 2E). To further examine the possible prolongation of formalin-induced pain by WT hevin, we quantified spontaneous pain in Phase 2+ (45–60 min) and found significant increase in Phase 2+ by WT hevin in male mice (*P* < 0.05 vs. hevin-ΔDE; Figure 2C), female mice (*P* < 0.05 vs. hevin-ΔDE; Figure 2D), and both sexes combined (*P* < 0.001 vs. hevin-ΔDE; Figure 2E). However, Phase 1 pain behavior was not affected by intrathecal hevin-ΔDE (10 μg) in both male and female mice (Figure 2, C–E). These results suggested that hevin is sufficient to induce mechanical allodynia in naive mice, potentiate central sensitization, and further enhance pain in neuropathic pain mice. Recent studies have shown sex dimorphism in spinal cord regulation of pain by glial cells and immune cells (29–31). We did not find sex differences in hevin-induced pain, as i.t. hevin potentiated formalin-induced pain (Figure 2, C–E) and induced robust mechanical allodynia in both males and females (Supplemental Figure 2A).

### Re-expression of hevin in spinal astrocytes reinstates neuropathic pain.

**Figure 3:**
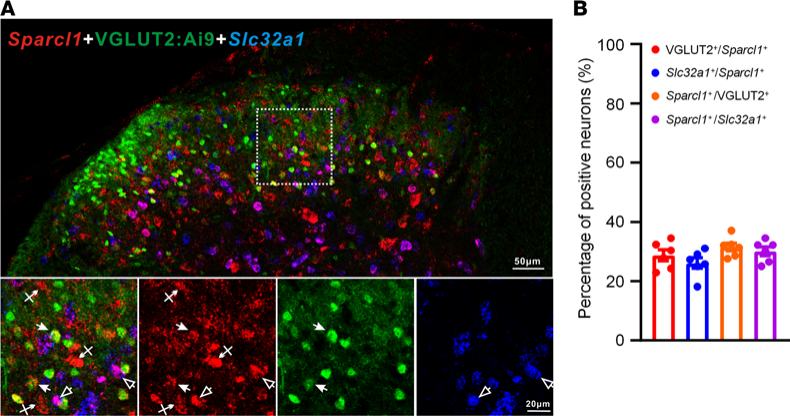
RNAScope images showing *Sparcl1* expression in excitatory and inhibitory neurons in the SDH of VGLUT2:Ai9 mice. (**A**) RNAScope images show colocalization of *Sparcl1* (red) with VGLUT2^+^ excitatory neurons (green) and *Slc32a1*^+^ inhibitory neurons (blue). Top, merged low-magnification image. Scale bar: 50 μm. Bottom, merged and single-channel images enlarged from the box in the top panel. Filled arrows show *Sparcl1*^+^/VGLUT2^+^ excitatory neurons, open arrows show *Sparcl1*^+^/*Slc32a1*^+^ inhibitory neurons, and arrows with cross show only Sparcl1^+^ cells. Scale bar: 20 μm. (**B**) Quantification of the percentage of *Sparcl1*^+^ cells expressing VGLUT2 or *Slc32a1* and the percentage of VGLUT2^+^ or Slc32a1^+^ cells expressing *Sparcl1* in the SDH. *n* = 6 spinal cord sections from 3 mice. Data are shown as mean ± SEM.

To investigate the cellular locations of hevin in the spinal cord, we examined *Sparcl1* (mouse hevin gene) expression in the SDH by RNAScope ISH. We conducted double staining of *Sparcl1* and *Slc32a1* (encoding vesicular inhibitory aa transporter) to localize hevin in inhibitory neurons. To localize hevin in excitatory neurons, we also performed double staining in VGLUT2:Ai9 mice. We found that *Sparcl1* is partly expressed in VGLUT2^+^ excitatory neurons and *Slc32a1*^+^ inhibitory neurons (Figure 3A). Quantitative analysis revealed that *Slc32a1* is evenly distributed in excitatory and inhibitory neurons of SDH (Figure 3B).

**Figure 4:**
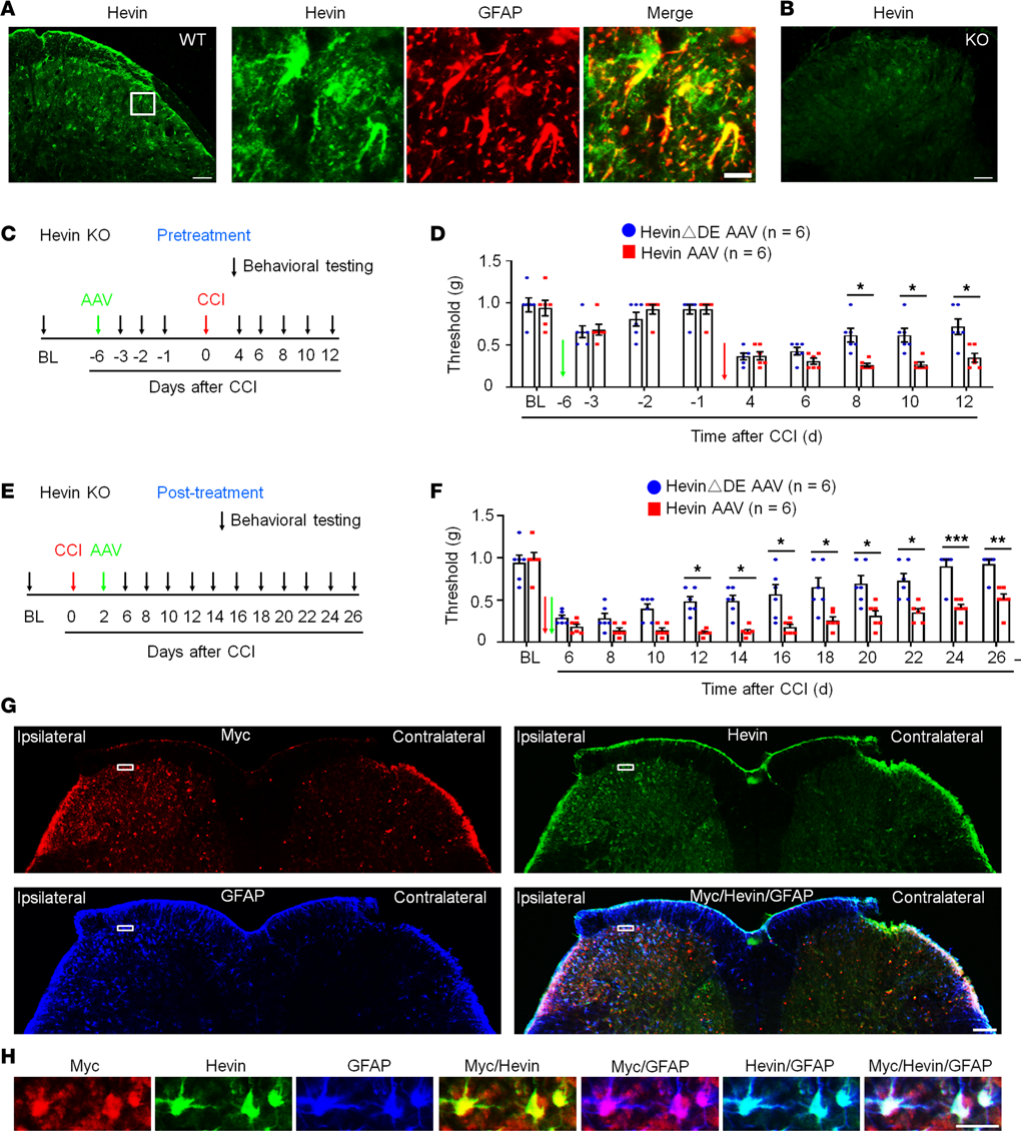
Expression of hevin in spinal astrocytes by intraspinal hevin-AAV reinstates neuropathic pain in hevin-KO mice. (**A**) Double immunostaining of hevin (green) and GFAP (red) in SDH. Note hevin is primarily colocalized with GFAP. Scale bars: 100 μm (left); 20 μm (right). The box is enlarged in the right panels. (**B**) Absence of hevin immunostaining in SDH in hevin-KO mice. Scale bar: 100 μm. (**C**) Paradigm for measuring mechanical allodynia in hevin-KO mice with intraspinal microinjection of hevin-AAV and hevinΔDE-AAV, given 6 days before CCI. (**D**) SDH microinjection of AAV-induced reduction in PWT in naive hevin-KO mice. After CCI, mechanical allodynia was significantly more prolonged in hevin-AAV–treated mice than hevinΔDE-AAV–treated mice. *n* = 6 mice/group. *P < 0.05, 2-way ANOVA followed by Bonferroni’s post hoc test. Green and red arrows indicate the time of virus injection and nerve injury, respectively. (**E**) Paradigm for measuring mechanical allodynia in hevin-KO mice with intraspinal microinjection of hevin-AAV and hevinΔDE-AAV, given 2 days after CCI. (**F**) SDH microinjection of hevin-AAV, given after nerve injury, significantly enhanced and prolonged mechanical allodynia in hevin-KO mice vs. hevinΔDE-AAV–treated mice. *n* = 6 mice/group. *P < 0.05, **P < 0.01, ***P < 0.001, 2-way ANOVA followed by Bonferroni’s post hoc test. Arrows indicate the time of virus injection and nerve injury. (**G**) Triple immunostaining of Myc (red), hevin (green), and GFAP (blue) in SDH in hevin-KO mice, 24 days after the ipsilateral SDH hevin-AAV injection. Note hevin expression is absent in the contralateral SDH of hevin-KO mice. Scale bar: 100 μm. (**H**) Enlarged images in the box of F panel **G**, with additional merged images for Myc/hevin, Myc/GFAP, and hevin/GFAP. Note hevin^+^ astrocytes also Myc^+^/GFAP^+^ in superficial SDH. Scale bar: 20 μm. All data are shown as mean ± SEM.

IHC revealed that hevin is expressed by the majority of astrocytes (glial fibrillary acidic protein–positive [GFAP^+^]; Figure 4A) and some neurons (NeuN^+^; Supplemental Figure 3, A and B), but not by microglia (Iba1^+^; Supplemental Figure 3, C and D) in SDH. The specificity of the hevin Ab was validated by loss of hevin staining in SDH of hevin-KO mice (Figure 4B). Western blot result also confirmed hevin expression in WT-SDH, which was lost in SDH of KO mice (Supplemental Figure 3E; see complete unedited blots in the supplemental material). IHC also showed hevin expression in neurons and satellite glial cells of dorsal root ganglion (DRG) of WT mice, but this staining was absent in hevin-KO mice (Supplemental Figure 3, F and G).

To determine a specific role of hevin from spinal astrocytes in neuropathic pain, we evaluated whether hevin re-expression in SDH astrocytes would reinstate neuropathic pain in hevin-KO mice (Figure 1G). To this end, we conducted SDH microinjection of AAV2/5.GFAP.Hevin-MycHis (hevin-AAV) (Supplemental Figure 1B) (9) to re-express hevin in spinal astrocytes in hevin-KO mice. We also included AAV2/5.GFAP.HevinΔDE-MycHis virus as a negative control (hevinΔDE-AAV) (Supplemental Figure 1B). AAVs were injected to the ipsilateral SDH of KO mice either prior to CCI nerve injury (pretreatment) (Figure 4, C and D) or after CCI (after treatment) (Figure 4, E and F). The pretreatment in naive mice induced mild and transient reduction in PWT in both hevin-AAV and hevinΔDE-AAV groups, due to spine surgery, but this PWT change fully recovered 5 days after the surgery (Figure 4D). Following CCI, mice treated with hevinΔDE-AAV exhibited similar time course of PWT change as in hevin-KO mice (Figure 1G), and neuropathic pain began to recover at 8 days following CCI (Figure 4D). Strikingly, hevin-AAV–treated KO mice showed no sign of neuropathic pain recovery at 8–12 days after CCI, and mechanical allodynia was significantly prolonged compared with hevinΔDE-AAV–treated mice (*P* < 0.05; Figure 4D). Furthermore, treatment of hevin-AAV, given 2 days after CCI (Figure 4E), also significantly decreased PWT compared with the hevinΔDE-AAV mice (*P* < 0.05), displaying prolonged neuropathic pain at 26 days after CCI (Figure 4F). Thus, both pretreatment and after treatment of hevin-AAV in KO mice restore neuropathic pain by enhancing and sustaining mechanical allodynia after CCI.

We confirmed the specific expression of hevin in SDH astrocytes after the hevin-AAV injection in the spinal cord sections of hevin-KO mice. We used Abs against hevin and GFAP, as well as Myc (Figure 4, G and H), as hevin-AAV has a Myc-tag (Supplemental Figure 1B). At 24 days after the ipsilateral SDH hevin-AAV injection, we found many GFAP^+^ astrocytes in the ipsilateral SDH, which were also labeled for hevin and Myc (Figure 4G), especially laminae I–III, a critical region for the transmission of mechanical pain (32, 33). High magnification images further revealed that hevin^+^ astrocytes also expressed Myc and GFAP in superficial SDH (Figure 4H). We did not observe hevin expression in the contralateral SDH of hevin-KO mice (Figure 4G). As previously reported (34), CCI caused a dramatic increase in the number of GFAP^+^ cells (astrogliosis) in the ipsilateral SDH. Collectively, these behavioral and histochemical data suggest that re-expression of hevin in spinal cord astrocytes is sufficient to reinstate neuropathic pain.

### Hevin regulates NMDA-evoked pain and NMDA currents in SDH neurons.

**Figure 5:**
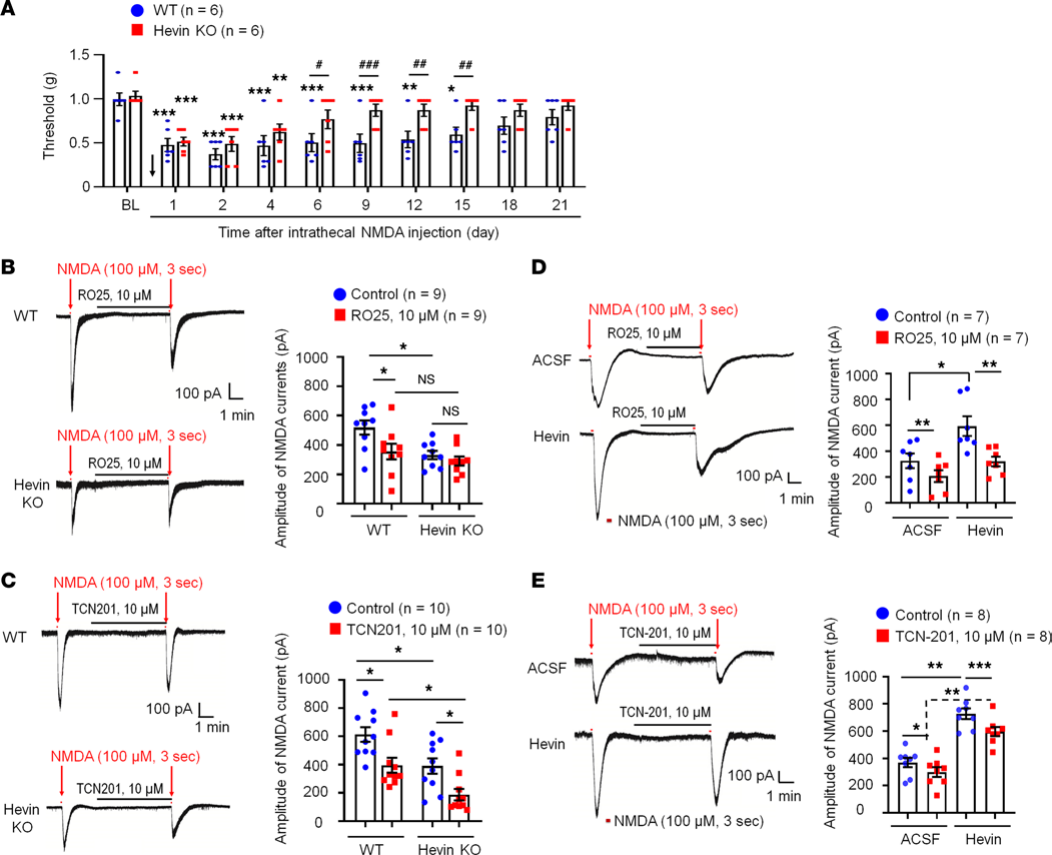
Hevin regulates NMDA-evoked pain and enhances NMDA currents in SDH neurons. (**A**) The duration of mechanical allodynia, induced by intrathecal injection of NMDA (3 nmol), is significantly shorter in hevin-KO mice than in WT mice. *n* = 6 mice/group. *P < 0.05, **P < 0.01, ***P < 0.001, compared with BL group; ^#^*P* < 0.05, ^##^*P* < 0.01, ^###^*P* < 0.001, 2-way ANOVA followed by Bonferroni’s post hoc test. Arrow indicates the time of NMDA injection. (**B** and **C**) Left: representative traces of inward currents in WT and hevin-KO mice, induced by NMDA (100 μM, 3 sec) via bath application. Note smaller NMDA currents in hevin-KO mice and different effects of RO25-6091 (GluN2B antagonist) and TCN-201 (GluN2A antagonist). Right: amplitude of NMDA-induced currents. *n* = 9, 10 neurons per group (shown in each column). *P < 0.05, 1-way ANOVA followed by Bonferroni’s post hoc test. (**D** and **E**) Left: representative traces of inward currents in ACSF- and hevin-treated (100 ng/mL, 4 min) spinal cord slices, induced by NMDA (100 μM, 3 sec) via bath application. Note different effects of Ro25-6091 and TCN-201. Right: amplitude of NMDA-induced currents. *n* = 7 neurons per group. *P < 0.05, **P < 0.01, ***P < 0.001, 1-way ANOVA followed by Bonferroni’s post hoc test. Data are shown as mean ± SEM.

Previously, we found that hevin strongly enhances NR2B subunit-containing NMDAR function by increasing NMDA currents in autoptic neuron-only cultures (9). Thus, here we tested the function of spinal NMDARs in WT and hevin-KO mice using behavioral and electrophysiological approaches. Spinal injection of NMDA (3 nmol, i.t.) induced persistent mechanical allodynia in WT mice, which lasted for more than 2 weeks and the mice recovered after 3 weeks (Figure 5A). The duration of NMDA-evoked mechanical allodynia was remarkably shortened in hevin-KO mice, showing a full recovery at 6 days after injection (Figure 5A).

We recorded NMDA-induced currents in spinal lamina IIo neurons via bath application of NMDA (100 μM, 3 sec) to spinal cord slices in WT and hevin-KO mice. Compared with WT neurons, hevin-KO neurons had significantly smaller NMDA-elicited currents (*P* < 0.05; Figure 5B). Since GluN2A and GluN2B containing NMDARs may play different roles in synaptic transmission and synaptic plasticity (5), we further tested the blocking effects of GluN2A antagonist TCN201 (10 μM) and GluN2B antagonist RO25 (10 μM) on NMDA currents in spinal cord slices from WT and hevin-KO mice. RO25 produced a significant inhibition of NMDA currents in WT neurons (31%, *P* < 0.05) but only evoked mild inhibition (12%, *P* > 0.05) of NMDA currents in hevin-deficient neurons (Figure 5B). In contrast, TCN (10 μM) produced significant and comparable inhibition of NMDA currents in both WT (26%, *P* < 0.05) and KO (26%, *P* < 0.05) neurons (Figure 5C). Therefore, we conclude that hevin deficiency primarily affects the GluN2B-mediated NMDA currents in lamina IIo neurons.

Next, we investigated whether perfusion of spinal cord slices with hevin is sufficient to enhance NMDA-evoked currents in WT mice. A brief exposure of SDH neurons to hevin (4 min, 100 ng/mL ≈ 0.14 nM) elicited a significant increase in NMDA-induced currents (Before: 450.3 ± 75.5 picoampere [pA]; After: 625.9 ± 87.3 pA; *P* < 0.05; Supplemental Figure 4A). Hevin-induced potentiation of NMDA currents was completely blocked by GluN2B antagonist RO25 (10 μM; Figure 5D). In contrast, GluN2A antagonist TCN (10 μM) produced a similar inhibition of NMDA currents in artificial CSF (ACSF) and hevin-treated neurons (19% vs. 18% inhibition, *P* < 0.05; Figure 5E). Hevin still evoked significant increase in NMDA currents after TCN-201 treatment (*P* < 0.05; Figure 5E). However, hevin had no effects on AMPA-evoked currents in lamina IIo neurons (Supplemental Figure 4B). We further assessed evoked excitatory postsynaptic currents (eEPSCs) using dorsal root stimulation. Hevin enhanced the NMDAR-mediated eEPSCs in lamina IIo neurons (Supplemental Figure 4C). Collectively, these data suggest that hevin is sufficient and required to regulate GluN2B/NMDAR–mediated currents in SDH neurons. This hypothesis was further supported by our behavioral observation that mechanical allodynia by intrathecal hevin was completely reversed by RO25 in both sexes (Supplemental Figure 2B). Consistent with a previous study (29), we did not find sex differences in NMDAR-mediated pain (Supplemental Figure 2B). Furthermore, hevin superfusion (100 ng/mL) increased spontaneous EPSC frequency and amplitude in lamina IIo neurons of spinal cord slices from CCI mice (Supplemental Figure 4D).

### Hevin-neutralizing Ab reduces inflammatory, postoperative, and neuropathic pain in WT mice.

**Figure 6:**
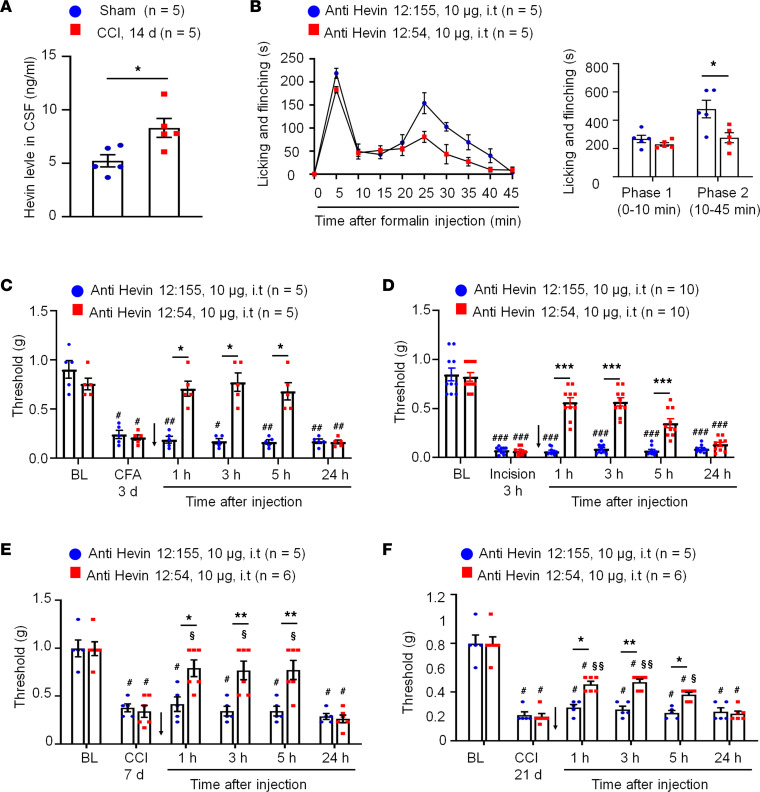
Anti-hevin monoclonal Ab 12:54 reduces inflammatory, postoperative, and neuropathic pain in WT mice. (**A**) ELISA analysis showing increased hevin level in the CSF 14 days after CCI. *n* = 5 mice/group. *P < 0.05, unpaired Student’s *t* test. (**B**) Left, time course of formalin-induced pain in WT male mice treated with intrathecal anti-hevin 12:155 monoclonal Ab (control Ab, 10 μg) or anti-hevin 12:54 monoclonal Ab (function blocking Ab, 10 μg). *n* = 5 mice per group. Right, formalin-induced Phase 1 and Phase 2 responses. *P < 0.05, unpaired Student’s *t* test. (**C**) Intrathecal injection of anti-hevin 12:54 Ab (10 μg), given 3 days after CFA injection, reduced CFA-induced mechanical allodynia for 5 hours. Arrows indicate the time of Ab injection. n = 5 mice/group. ^#^*P* < 0.05, ^##^*P* < 0.01 versus corresponding BL group; *P < 0.05 versus anti-hevin 12:155 group, 2-way ANOVA followed by Bonferroni’s post hoc test. (**D**) Intrathecal injection of anti-hevin 12:54 Ab (10 μg) given 3 hours after plantar incision, reduced incision-induced mechanical allodynia for 5 hours in male and female mice. Arrows indicate the time of Ab injection. *n* = 10 mice/group. ^###^*P* < 0.001 versus corresponding BL group; ***P < 0.001 versus anti-hevin 12:155 group, 2-way ANOVA followed by Bonferroni’s post hoc test. (**E** and **F**) Intrathecal injection of anti-hevin 12:54 Ab (10 μg), given 7 days **E** and 21 days **F** after nerve injury, reduced CCI-induced mechanical allodynia for 5 hours. Arrows indicate the time of Ab injection. *n* = 5–6 mice/group. ^#^*P* < 0.05 versus corresponding BL group; ^§^*P* < 0.05, ^§§^*P* < 0.01 versus corresponding baseline at CCI 7 days or CCI 21 days; *P < 0.05, **P < 0.01, 2-way ANOVA followed by Bonferroni’s post hoc test. Data are shown as mean ± SEM.

To examine if hevin is secreted from the mouse spinal cord, we measured hevin levels in the CSF using a mouse hevin-specific ELISA kit. We detected a high basal level of hevin secretion (~5 ng/mL) in the CSF of naive mice (Figure 6A). To determine the role of secreted hevin in inflammatory and neuropathic pain, we tested a monoclonal Ab against mouse hevin, which we previously characterized (9). The monoclonal Ab recognizes an epitope mapping to the aa 368–419 of hevin (anti-hevin 12:54; Supplemental Figure 1C) and this Ab blocks hevin’s synaptogenic function, likely by interfering with hevin’s ability to bind Nrx1a and/or NL1B (9). Another hevin Ab recognizing a different epitope (anti-hevin 12:155; Supplemental Figure 1C) does not impact hevin’s synaptogenic activity, which we used as our control. Intrathecal injection of anti-hevin 12:54 (10 μg) significantly reduced formalin-induced Phase II pain compared with anti-hevin 12:155-treated mice (*P* < 0.05; Figure 6B). We also tested these 2 Abs in a chronic inflammatory pain model (induced by CFA; Figure 6C) and a postoperative pain (induced by plantar incision; Figure 6D). Intrathecal injection of anti-hevin 12:54 (10 μg) significantly reduced mechanical allodynia in the CFA model (*P* < 0.05 at 1, 3, and 5 hours; Figure 6C) and incision model (*P* < 0.001 at 1, 3, and 5 hours; Figure 6D), compared with anti-hevin 12:155-treated mice. Furthermore, intrathecal injection of anti-hevin 12:155 (10 μg), but not anti-hevin 12:155 (10 μg), rapidly (< 1 hour) reversed mechanical allodynia for more than 5 hours in CCI mice, in both early phase (7 days after CCI; Figure 6E) and late phase (21 days after CCI; Figure 6F) of neuropathic pain. ELISA analysis showed that CCI resulted in a significant increase in CSF-hevin levels (Sham: 5.24 ± 0.57 ng/mL; 14 days after CCI: 8.32 ± 0.89 ng/mL; *P* < 0.05; Figure 6A). These results suggest that 1) hevin is secreted in physiological and pathological conditions and 2) targeting secreted hevin with a neutralizing Ab can potently alleviate inflammatory and neuropathic pain.

### Hevin is increased in human CSF following intracranial surgery.

**Figure 7:**
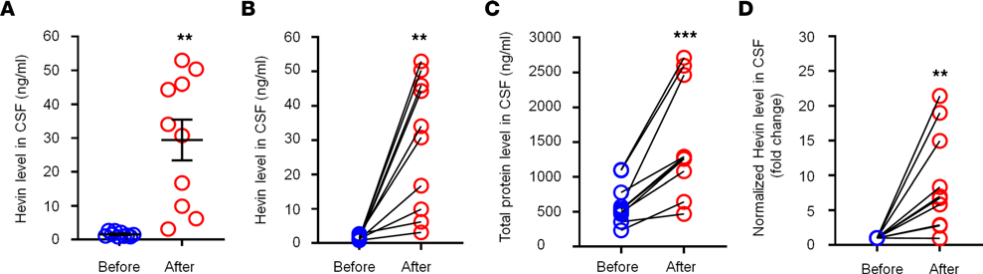
Intracranial surgery increases hevin levels in human CSF samples. (**A** and **B**) ELISA analysis showing increased hevin levels in human CSF 12 hours after intracranial surgery. The same data were presented unpaired **A** and paired **B**. (**C**) Bicinchoninic acid (BCA) protein assay showing total protein level increased in human CSF 12 hours after surgery. (**D**) Fold changes of normalized hevin level in human CSF 12 hours after surgery, normalized to total protein changes. *n* = 10 patients. **P < 0.01, ***P < 0.001, paired Student’s *t* test. Data shown as mean ± SEM.

To explore the translational potential of these findings, we next assessed secreted hevin levels in human CSF samples collected prior to and 12 hours after intracranial surgery (e.g., otolaryngology procedures) (35), using a human hevin-specific ELISA kit. In the human CSF, we were able to detect a basal secretion of hevin, ranging from 0.8–2.7 ng/mL (Figure 7A). Intracranial surgery resulted in a rapid and dramatic increase in CSF hevin levels, ranging from 3.1–52.9 ng/mL at 12 hours (Figure 7A). Strikingly, hevin showed marked increases in all the CSF samples we analyzed, ranging from a 2.3- to 50.9-fold increase (*P* < 0.01; Figure 7B). We also measured total protein levels in these CSF samples and observed mild but significant increases, ranging from a 1.3- to a 4.3-fold increase (*P* < 0.001; Figure 7C). After normalization with respective protein changes, CSF-Hevin levels still exhibited a significant increase (9.0-fold, *P* < 0.01; Figure 7D). These results suggest that human neurosurgery selectively increased the CSF section of hevin, beyond the postoperative increase in CSF total protein levels.

## Discussion

Despite recent progress in demonstrating gliopathy in the pathogenesis of pain (15) and the previous studies showing an essential role of spinal TSP4 and cortical TSP1 in neuropathic pain sensitization (24, 25, 36), our knowledge is still limited regarding the specific mediators secreted by astrocytes that can drive neuropathic pain. We employed both loss-of-function and gain-of-function approaches to demonstrate a critical role of astrocytic hevin in driving neuropathic pain. Our data showed that nerve injury-induced mechanical allodynia in the maintenance phase, but not the induction phase, is impaired after hevin deficiency, in further support of the notion that astrocytes are crucial for the maintenance of neuropathic pain (15). Nerve injury induces marked and long-lasting astrogliosis in SDH that is correlated with the time course of neuropathic pain (6, 18). It has been shown that hevin is expressed by reactive astrocytes in the brain (37). We demonstrate that re-expression of hevin in SDH reactive astrocytes in hevin-KO mice is sufficient to reinstate neuropathic pain after nerve injury. Furthermore, intrathecal injection of hevin produced persistent mechanical allodynia in WT mice of both sexes.

Mechanistically, we demonstrated that hevin induces central sensitization and mechanical pain through regulation of GluN2B containing NMDARs in SDH neurons, in agreement with our previous report that hevin is crucial for the formation of thalamocortical connections in the visual cortex via specific regulation of GluN2B during development (8). Thus, hevin-induced mechanical pain was completely blocked by the GluN2B antagonist RO25. As expected, hevin deficiency resulted in a significant reduction of NMDA currents in SDH neurons through adulthood. Interestingly, physiological pain in adult mice at the baseline is unaltered in hevin-KO mice, suggesting a specific contribution of NMDARs to synaptic plasticity in pathological pain (3). Although we did not find significant change in AMPA-induced currents in SDH neurons of hevin-KO mice, a recent study shows that spinal hevin also mediates membrane trafficking of GluA1-containing AMPA receptors in remifentanil-induced postoperative hyperalgesia (38). Our findings strongly suggest that hevin-induced synaptic plasticity not only occurs during development but also manifests in pathological and neurodegenerative conditions such as neuropathic pain.

Our data also showed that hevin is highly secreted in mouse CSF and this secretion is further increased after nerve injury. Notably, CSF-hevin changes are clinically relevant. We found a rapid and substantial increase of hevin in human CSF samples 12 hours after painful neurosurgical procedures (35). Neurosurgery/otolaryngology procedures are also associated with an increase in the CSF tau levels that have been implicated in the development of dementia (35). It is of great interest to examine CSF-hevin levels in a general surgery population. Thus, CSF hevin could potentially serve as a biomarker for pain following neurosurgery or general surgery, an important question for evaluation in future studies.

It is well-established that overactivation of GluN2B causes neurotoxicity (39). Thus, high levels of hevin production and secretion in the CNS are not only associated with pain but may also underlie surgery-induced neurocognitive injury such as delirium (40) by producing GluN2B-mediated neurotoxicity. Future studies are warranted to evaluate the involvement of hevin in neurocognitive function under physiological and pathological conditions. Importantly, we have demonstrated that neutralizing secreted hevin with a monoclonal Ab could effectively alleviate inflammatory and neuropathic pain. Thus, targeting secreted hevin may offer new therapeutics to manage chronic pain, postoperative neurocognitive injury, and neurogenerative diseases.

There are several imitations in this study. First, despite substantial increases of hevin in all the human CSF samples generated in the acute phase of intracranial surgery, the sample size of this study is small, and it is difficult to associate increases in Hevin abundance with pain in patients. It is also possible that an increase in CSF hevin is a general response to surgery or acute stress. Future studies are needed to collect more CSF samples at multiple time points of surgeries or other painful diseases to correlate hevin levels with acute pain and chronic pain. Second, hevin is produced not only by astrocytes but also by a subset of neurons in the spinal cord and DRG. It remains to be identified whether hevin in the CSF is mainly derived from astrocytes. Deletion of *Sparcl1* in different cell types using conditional KO mice will help to address this issue. Finally, hevin may regulate synaptic plasticity and pain via non-NMDARs, such as AMPA receptors (38). In addition to neuronal modulation, hevin may regulate microglia activation that has been strongly implicated in chronic pain development (41, 42). It will be of great interest to study hevin-mediated neuron-glial and glia-glial interactions in acute and chronic pain.

## Methods

### Reagents.

We purchased formalin, carrageenan, CFA, and NMDA from Sigma-Aldrich; TCN201 (GluN2A antagonist; catalog 4154) and RO25-6981 (GluN2B antagonist; catalog 1594) from Tocris; and recombinant hevin from R&D Systems (catalog 4547-SL). Purified recombinant hevin and hevin-ΔDE (lacking aa 351–440) proteins, anti-hevin monoclonal Ab 12:155, and anti-hevin monoclonal Ab 12:54 were from the laboratory of CE at Duke University Medical Center.

### Mice.

Hevin-KO mice were from the laboratory of CE (9), and control SVE/129 mice (aged 8–12 weeks of both sexes) were obtained from Taconic Biosciences. These mouse lines were maintained at the Duke University animal facility. We also used CD1 mice (Charles River Laboratories, 8–10 weeks) of both sexes for comparing hevin’s or anti-hevin’s effects on mechanical pain. VGLUT2:Ai9 mice were made by crossing Ai9 mice (The Jackson Laboratory, catalog JAX:007909) with vGlut2 flox mice (JAX:033688) for the RNAScope experiment. All animals were housed under a 12-hour light/dark cycle with food and water available ad libitum. Sample sizes were estimated based on our previous studies for similar types of behavioral and electrophysiological analyses (5, 19). Animals were randomly assigned to each experimental group.

### Mouse models of pain, CSF collection, and intrathecal injection.

To produce inflammatory pain, diluted formalin (5%, 20 μL), carrageenan (1.5 %, 20 μL), or CFA (20 μL) was injected into the plantar surface of a hindpaw (5). Neuropathic pain was induced by CCI as previously published (19). In brief, after the left sciatic nerve was exposed, 3 ligatures (LOOK 6-0 Silk, catalog SP102) were placed around the nerve proximal to the trifurcation with 1 millimeter between each ligature. The ligatures were loosely tied until a short flick of the ipsilateral hind limb was observed. Mice in the sham group received surgery identical to those described but without nerve ligation. Mouse CSF was collected from the cisterna magna 14 days after CCI (34). Plantar incision surgery was performed as previously described (43). A 5 mm longitudinal incision was made by #11 blade through the skin and fascia of the plantar foot, starting 2 mm from the proximal edge of the heel. For intrathecal injection, spinal cord puncture was made with a 30G needle between the L5 and L6 levels to deliver reagents (10 μL) to the CSF (34).

### Spinal injection of AAV2/5 virus.

As in our previous report (9), full-length hevin cDNA was cloned into the pZac2.1 gfaABC1D-Cyto-GCaMP3 viral vector (Addgene, plasmid, catalog 44331), between the NheI and NotI restriction sites, replacing the GCaMP3 sequence. AAV2/5 particles were packaged and synthesized by the Penn Vector Core facility (University of Pennsylvania). Intraspinal injections were performed as described previously (44). In brief, hevin-KO mice were deeply anesthetized by s.c. injection of ketamine (100 μg/g) and xylazine (10 μg/g). Paraspinal muscles around the left side of the interspace between T13 and L1 vertebrae were removed, and the dura mater and the arachnoid membrane were carefully incised using the tip of a 30G needle to make a small window to allow a glass micropipette (diameter of 80 μm) insert directly into the SDH. The microcapillary was inserted with AAV2/5 virus solution through the small window (approximately 500 μm lateral from the midline) and inserted into the SDH (250 μm in depth from the surface of the dorsal root entry zone). Each mouse was given a single injection (0.7 μL, 1 x 10^12^ GC/mL) of hevin-AAV.SV40 virus or hevinΔDE-AAV.SV40 virus.

### Behavioral testing.

Animals were habituated to the environment for at least 2 days before the testing. The room temperature and humidity remained stable for all experiments. All the behaviors were tested blindly. Key behavioral studies were repeated by different lab members. For testing spontaneous pain in formalin model, we measured the time (seconds) mice spent on licking or flinching the affected paw every 5 minutes for 45 or 60 minutes. For testing mechanical sensitivity, we confined mice in boxes placed on an elevated metal mesh floor and stimulated their hindpaws with a series of von Frey hairs with logarithmically increasing stiffness (0.02–2.56 g, Stoelting), presented perpendicularly to the central plantar surface. We determined the 50% PWT by Dixon’s up-down method (45). Thermal sensitivity was assessed by tail immersion, hot plate, and radiant heat tests. For the tail immersion test, the lower 5.0 cm portion of the tail was dipped in hot water at 48°C or 52°C and the tail flick latency was recorded with a cut-off time of 20 seconds. For the hot plate test, mice were placed on the hot plate at 50°C or 56°C and the reaction time was scored when the animal began to exhibit signs of pain avoidance such as jumping or paw licking. Animals that did not respond to the noxious heat stimulus after 30 seconds were removed from the plate. For the radiant heat test, Hargreaves apparatus (IITC Life Science) was used, and the basal paw withdrawal latency was adjusted to 9–12 seconds with a cutoff of 20 seconds to prevent tissue damage.

Spontaneous and ongoing pain was assessed by the CPP test. We used a single trial conditioning protocol to measure the CPP. All mice underwent a 3-day preconditioning habituation and animal behavior was videorecorded. Analyses of the preconditioning (baseline) behavior showed no pre-existing chamber preference. On the conditioning day, mice received the vehicle (PBS, 10 μL, i.t.) control paired with a randomly chosen chamber in the morning and clonidine (10 μg in 10 μL PBS, i.t.) paired with the other chamber 4 hours later. Chamber pairings were counterbalanced. On the test day (3 weeks after CCI), 20 hours following the afternoon pairing, mice were placed in the CPP box with access to both chambers and the behavior was recorded for 15 minutes and analyzed by ANY-maze software for chamber preference.

### ISH, immunofluorescence, and imaging.

After appropriate survival times after nerve injury, mice were deeply anesthetized with isoflurane and perfused through the ascending aorta with PBS and then followed by 4% paraformaldehyde. The L4–L5 spinal cord segments and L4–L5 DRGs were removed and postfixed in the same fixative overnight. Spinal cord (30 μm, free floating) and DRG tissue sections (14 μm) were cut in a cryostat. In accordance with the manufacturer’s instructions of RNAScope system (Advanced Cell Diagnostics), ISH was performed by the Multiplex Fluorescent Kit v.2. We used probes directed against mouse *Sparcl1* (catalog 424641, NM_ 010097.4) and mouse *Slc32a1* (catalog 319191-C3, NM_ 009508.2) on the spinal cord sections from VGLUT2:Ai9 mice. For IHC, the sections were blocked with 2% goat or donkey serum for 1 hour at room temperature and then incubated overnight at 4°C with the following primary Abs: anti-hevin (rat anti-hevin monoclonal 12:155, 1 μg/mL, from CE’s lab), anti-NeuN (mouse, 1:1000; Millipore, catalog MAB377), anti-GFAP (mouse, 1:1000; Millipore, catalog MAB360), anti–IBA-1 (rabbit, 1:1000; Wako Chemicals, catalog 019-19741), and anti-Myc (mouse, 1:1000; Cell Signaling, catalog 2276). Tissue sections were then stained by cyanine 3(Cy3)^–^, cyanine 5(Cy5)^–^ and/or FITC-conjugated secondary Abs (1:400; Jackson ImmunoResearch) for 2 hours at room temperature. The stained sections were examined with a Nikon fluorescence microscope, and images were captured with a CCD Spot camera. For high resolution images, sections were also examined under a Zeiss 510 inverted confocal microscope. Quantification of RNAScope images was conducted in 5 spinal cord sections from 2 mice.

### Patch clamp recordings in spinal cord slices.

As previously reported (19), we removed a portion of the lumbar spinal cord (L4–L5) from young mice (4–6 weeks old) under urethane anesthesia (1.5–2.0 g/kg, i.p.) and kept the spinal cord segments in pre-oxygenated ice-cold ACSF. Transverse slices (400 μm) were made on a vibrating microslicer and the slices were transferred to ACSF saturated with 95% O_2_ and 5% CO_2_ at 26^o^C for at least 1 hour prior to experiment. The ACSF contains (in mM): NaCl 125, KCl 2.5, CaCl_2_ 2, MgCl_2_ 1, NaH_2_PO_4_ 1.25, NaHCO_3_ 26, and glucose 25. The whole cell patch-clamp recordings were conducted from lamina IIo neurons in voltage clamp mode. Lamina IIo interneurons are predominately excitatory, make direct connections with lamina I projection neurons, and play a critical role in transmitting mechanical pain (32, 33). Patch pipettes were prepared from thin-walled, borosilicate, glass-capillary tubing (1.5 mm outside diameter; World Precision Instruments) and filled with an internal solution containing following (in mM): K-gluconate 135, KCl 5, CaCl_2_ 0.5, MgCl_2_ 2, EGTA 5, HEPES 5, and ATP-Na_2_ 5. Data were acquired using an Axopatch 700B amplifier and were low pass filtered at 2 kHz and digitized at 5 kHz. NMDA currents were recorded in IIo neurons by perfusing spinal cord slices with 100 μM NMDA for 3 seconds in ACSF with TTX (5 μM). To measure NMDAR-mediated evoked EPSCs (NMDA-eEPSCs) in lamina IIo neurons, dorsal root (~ 5 mm) was stimulated through a suction electrode. NMDA-eEPSCs were pharmacologically isolated in Mg^2+^-free ACSF containing CNQX (10 μM), strychnine (2μm), and picrotoxin (100 μM). Neurons were voltage clamped at –40 mV and NMDA-eEPSCs were evoked at 0.05 Hz. QX-314 (5 mM) was added to the pipette solution to prevent discharge of action potentials. Spontaneous EPSCs were also recorded in lamina IIo neurons at holding potential of –70 mV. Synaptic strength was quantified by the peak amplitudes of eEPSCs. Data were collected with pClamp version 10 software and analyzed using Clampfit version 10.

### Western blot.

SDH protein samples were prepared as we previously demonstrated (46), and 30 μg of proteins were loaded for each lane and separated on SDS-PAGE gel (4%–15%; Bio-Rad). After the transfer, the blots were incubated overnight at 4°C with anti-hevin (rat anti-hevin monoclonal 12:155, 1 μg/mL). These blots were further incubated with HRP-conjugated secondary Ab, developed in ECL solution (Pierce), and the chemiluminescence was revealed by Bio-Rad ChemiDoc XRS for 1–5 minutes.

### Human CSF collection.

Human CSF was collected from the patients enrolled in the Markers of Alzheimer’s disease after Propofol vs. Isoflurane Anesthesia (MAD-PIA) trial. MAD-PIA was a prospective randomized trial registered with http://www.clinicaltrials.gov (NCT01640275) on June 20, 2012, by Miles Berger (35). Human CSF samples (10 mL) were obtained from the lumbar drain (AccuDrain; Integra Neurosciences, catalog INS-8400) at the time of drain placement (0 hours) and 10–12 hours after intracranial surgery.

### ELISA.

The mouse hevin ELISA kit was purchased from LifeSpan BioSciences (catalog LS-F12654). The human hevin ELISA kit was from Cloud-Clone Corp (catalog SEM267Hu). ELISA was conducted in mouse CSF samples (10 μL) and human CSF (10 μL) according to the manufacturer’s instructions. The standard curve was included in each experiment.

### Statistics.

All the data were expressed as the mean ± SEM, as indicated in the figure legends. Statistical analyses were completed with Prism GraphPad 8.0. Biochemical and behavioral data were analyzed using 2-tailed Student’s *t* test (2 groups, unpaired or paired) or 2-way ANOVA followed by Bonferroni’s post hoc test. Electrophysiological data were tested using 1-way ANOVA (for multiple comparisons) or 2-way ANOVA (for multiple time points) followed by Bonferroni’s post hoc test or Student’s *t* test (2 groups, unpaired or paired). The criterion for statistical significance was *P* < 0.05.

### Study approval.

All the animal procedures were conducted in accordance with the NIH Guide for the Care and Use of Laboratory Animals and approved by the IACUC of Duke University. The human CSF study was approved by the Duke University Institutional Review Board.

## Author contributions

GC, CE, and RRJ designed the research. GC, JX, HL, and XL performed the experiments and data analysis. SKS and JJR prepared critical reagents and participated in project discussions. MLJ, JPM, and MB provided human CSF samples and contributed to the related experiments. RRJ and CE supervised the project. GC and RRJ wrote the manuscript, and all other authors edited the paper.

## Supplementary Material

Supplemental data

## Version 1 10/18/2022

In-Press Preview

## Version 2 12/08/2022

Electronic publication
