# Supplementary material for: Hevin/Sparcl1 drives pathological pain through spinal cord astrocyte and NMDA receptor signaling
Source: JCI Insight. 2022 Dec 8;7(23):e161028. doi: 10.1172/jci.insight.161028 (PMC9746899; doi:10.1172/jci.insight.161028)
Supplement: Supplemental data [file jciinsight-7-161028-s130.pdf]

Supplemental Figures

Gang Chen, Jing Xu, Hao Luo, Xin Luo, Sandeep K. Singh, Juan J. Ramirez, Michael L. James, Joseph P. Mathew, Miles Berger, Cagla Eroglu, and Ru-Rong Ji

Supplemental Figure 1.

Supplemental Figure 2.

Supplemental Figure 3.

Supplemental Figure 4.

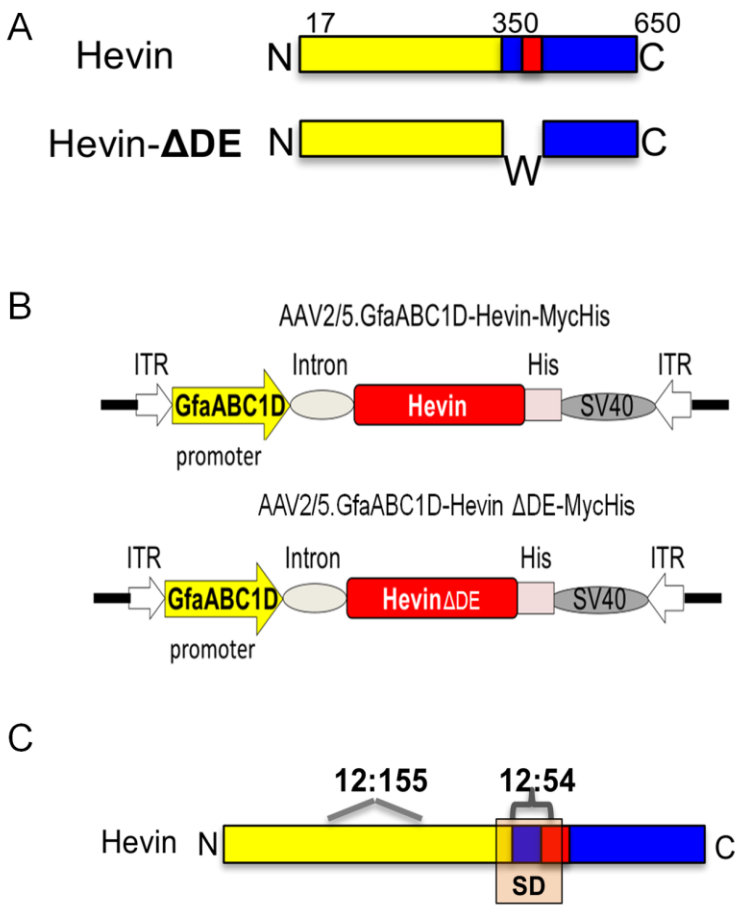

Supplemental Figure 1. Schematic of hevin protein (A), hevin-AAV (B), and mapped epitopes (c) for anti-hevin monoclonal antibodies.

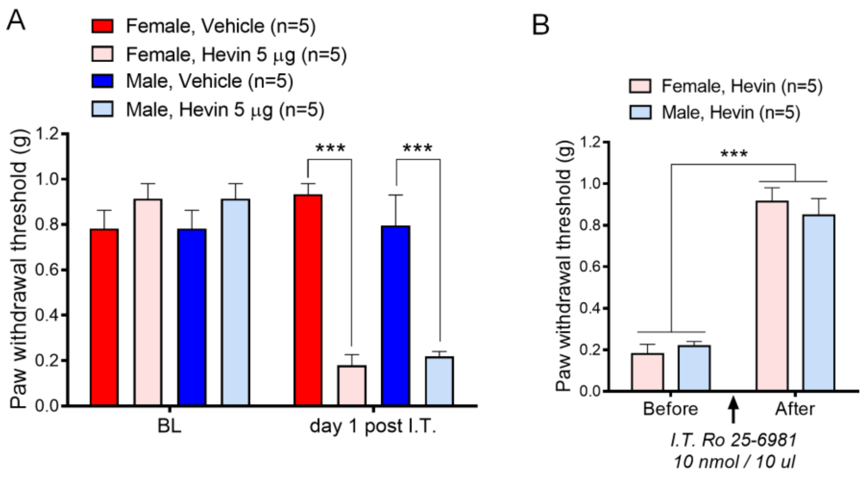

Supplemental Figure 2. Hevin and NR2B activation drive mechanical pain in both sexes.

(A) Intrathecal injection of purified WT hevin (5 μg, i.t.) induced robust mechanical allodynia in both WT naïve male and female mic for more than 1 day. n = 5 mice per group. ****P*<0.001, Two-Way ANOVA followed by Bonferroni posthoc test. Data shown as mean ± SEM. (B) Intrathecal hevin-induced mechanical allodynia was completely reversed by Ro25-6091 (GluN2B antagonist) in both WT male and female mice. n = 5 mice per group. ****P*<0.001. Two-Way ANOVA followed by Bonferroni posthoc test. Data shown as mean ± SEM.

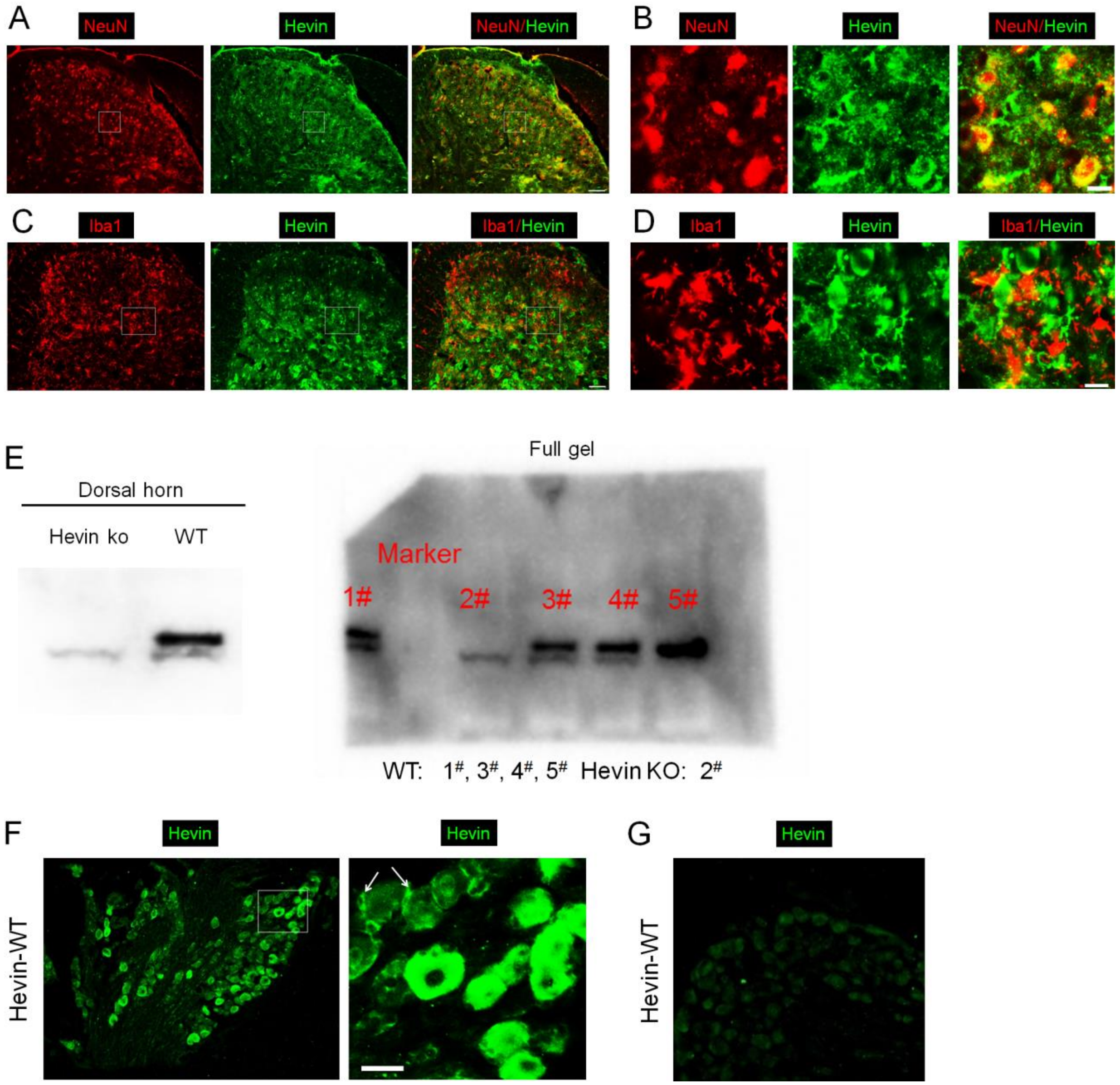

Supplemental Figure 3. Hevin expression in SDH and DRG of WT and KO mice.

(A, B) Double immunostaining of hevin (green, A) and the neuronal marker NeuN (red, B) in the spinal cord dorsal horn in mice. (C, D) Double immunostaining of hevin (green, C) and microglial marker Iba1 (red, D) in the spinal cord dorsal horn in mice. Note hevin is expressed by some neurons ($NeuN^+$) but not microglia ($Iba1^+$). Right images are enlarged images of the left image. Scale bars: 50 μm (left) and 10 μm (right). (E) Western blot showing hevin expression in spinal dorsal horn of WT mice and KO mice. Right, uncut full gel of the left blot. (F) Hevin expression in DRG neurons and satellite glial cells (indicated by arrows) of WT mice. Right, enlarged box of left. Scale, 20 μm. (G) Hevin expression in DRG is lost in hevin KO mice.

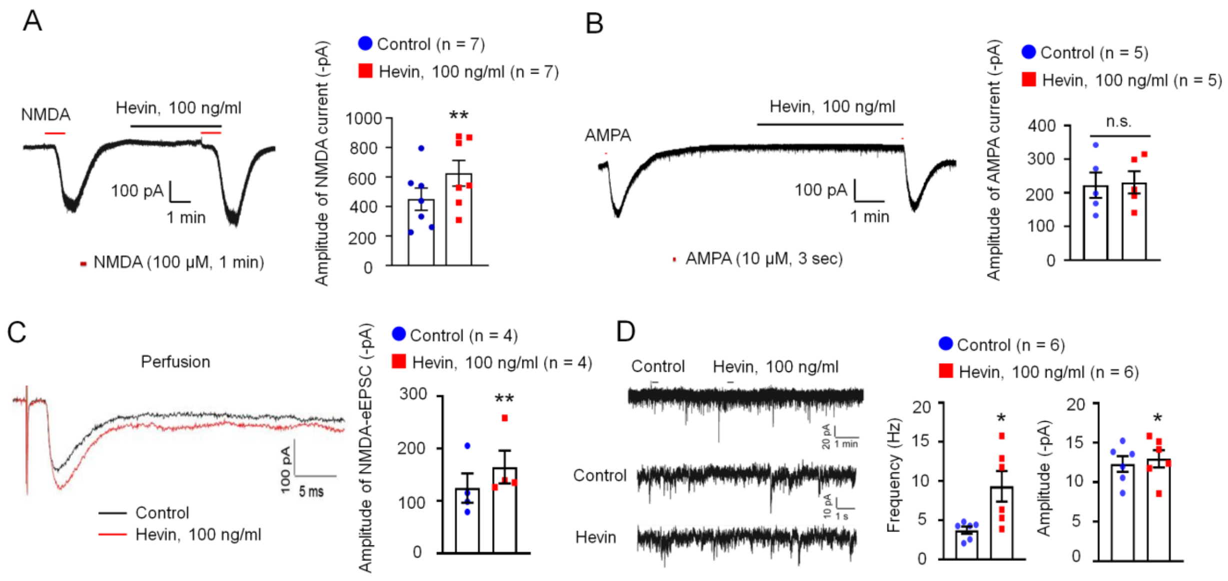

Supplemental Figure 4. Electrophysiological characterization of hevin's actions in lamina IIo neurons of spinal cord slices.

(A, B) Acute effects of hevin (100 ng/mL, 5 min) on NMDA or AMPA-induced current in lamina II neurons. Note hevin enhanced the NMDA-induced currents but no effects on AMPA-evoked currents in lamina IIo neurons. n = 7 or 5 neurons per group (shown in each column). ***P* < 0.01, paired t-test, n.s., not significant. Data shown as mean ± SEM. (C) Effects of hevin (100 ng/mL) on NMDA-eEPSC in lamina IIo neurons evoked by DR stimulation. Note hevin also enhanced the NMDA receptor–mediated eEPSCs. n = 4 neurons per group. ***P* < 0.01, paired t-test. Data shown as mean ± SEM. (D) Hevin superfusion (100 ng/ml) increased spontaneous EPSC frequency and amplitude in lamina IIo neurons of spinal cord slices from CCI mice. n = 6 neurons per group. **P*<0.05, paired t-test. Data shown as mean ± SEM.
